# Monthly Summary

**Published:** 1885-07

**Authors:** 


### Monthly Summary.

"Death from Inhaling Nitrous Oxide."—Dr. A. J. Shurtleff, a dentist practising in Naitch, Mass , and having an office also in Boston, at the Hotel Roylston, was found dead in his Boston office on the evening of February 26th, under circumstances which show that his death was caused by inhaling that form of nitrous oxide known as "Mayo's Vegetable Anæsthetic," which we have been informed is simply nitrous oxide that has been washed through some solution of vegetable compounds.

The janitor of the building upon being informed by the night watchman that Dr. S. had not gone out of his office, at about 10 o'clock p. m. entered the room and found the doctor lying upon the floor dead. The gas cylinder was lying upon the floor close by his head, completely empty of gas; the tube, detached from the inhaler, was held in the left hand; with one end of it held tightly between the teeth, the other of course attached to the cylinder. The right hand held the wheel wrench, close to, but disconnected from the cylinder, The face of the deceased was very much discolored, but the body was not cold. The physician summoned expressed the opinion that life had been extinct for about an hour. The last time the doctor was seen alive was about 6 o'clock, when the janitor opened the office door and found a gentleman friend with him. That friend says that about 5 o'clock he, at the doctor's request, gave him a little gas, the doctor telling him how to do it. This friend went away soon after 6 o'clock and left the doctor, as he says, all right. It is not supposed that it was a case of suicide, as there is no known reason for such a theory. The doctor had been complaining of a tooth-ache during the day, but it is not probable he took gas for that reason. Once, weeks before, the janitor had entered the office and found the doctor in his operating chair and in the act of giving himself gas. As the doctor was at times addicted to the too free use of spirituous liquors, the more probable theory is that he took gas when not in just the condition of mind to judge rightly in regard to it, and died as the result of carlessness on his own part in taking the gas for the pleasurable sensation it gave him. When found by the janitor in the act of giving himself gas he was slightly under the influence of something besides gas, and there is every reason to believe he had "been taking something previous" to the fatal attempt to administer gas to himself. No dentist should ever undertake to administer an anæsthetic to himself under any circumstances. The writer once found a Dental student in an anæsthetized condition with the inhaler still in his mouth and the gas turned on, and in all probability in a few minutes more death would have been the result of that student's experiment with gas "to see what it was like." There are many cases on record of persons dying from the attempt to put themselves to sleep with chloroform. Dr. Shurtleff was about 40 years old, and had been in practice nearly 20 years, and was considered a good and successful practitioner.—*Archives of Dentistry.*

---

SOME MODIFICATIONS OF THE ANTISEPTIC METHOD.—The *Annals of Surgery* for July contains the following abstract of Prof. Mikulicz' report of his last three year's work at Cracow. In practice solutions of bi-chloride are used at least ten times the strength which experiment has seemed to indicate as sufficient. Koch, however, pointed out that the weaker solutions were adequate only where all the disinfectant in solution could exert its full action, and that in the case of sublimate other strengths would have to be used for disinfection of fluids rich in albuminates and sulphur, or other compounds forming insoluble combinations with mercury. In wounds, the formation of albuminate of mercury immediately weakens the solution, so that it may be reduced from one of the strongest to a very moderate antiseptic.

For the prophylactic portion of wound-antisepsis—disinfection of sponges, tubes, sutures, operator's hands and part to be operated—such objection does not hold. Sublimate is incomparable, its value being increased by the rapidity of its action. It is not suitable for metal instruments, since they are attacked by it. During and after completion of an operation, on the other hand, we have to deal with albuminous secretions, a very varying factor. Experience, rather than experiments, must guide us here. Koch laid down the rule that the disinfecting solution should contain sufficient bichloride, that 1 part in 5,000 remain free. K. found that river water required 1:2000, bilge-water 1:1000, and putrid blood 1:400. Mikulicz experimented with defibrinated ox-blood and spring-water. Sublimate 1:1000 retarded and dimished the development of micro-organism· weaker solutions not perceptibly; while only a strength of 1:400-500 completely prevented their appearing. Comparative tests showed that it only needed about double this strength of carbolic to give as good results. Schill & Fischer found that 1-5 of sublimate solution, mixed with an equal volume of tuberculous sputum, quite failed to disinfect it, while a 5 per cent. carbolic sufficed. Since the excretion of pathogenic micro-organisms usually occurs in connection with albuminous material, sublimate is probably less trustworthy for such hygienic disinfection than carbolic His own tests with bichloride simply bore on the question of impeding the development of, rather than destroying germs, since the latter would require too irritating solutions. Mercury albuminate itself is, however, not entirely inactive, and does not readily putrify.

There are, therefore, good reasons for considering carbolic a far more constant and trustworthy wound antiseptic than sublimate, although the latter is the stronger agent. If sublimate is used in preparing for an operation and full precautions be taken, then even pure water (0.6 per cent. salt solution) may answer for wound irrigation (V. Jan. No. p. 88). In septic puerperal conditions, it is questionable whether sublimate should be used since either strong solutions or frequent irrigation may prove toxic. As a wound dressing iodoform, thymol, etc., are not inferior to bichloride, and less dangerous. Again, sublimate, contrary to general impression, is volatile. Kratschemer found that sublimated gauze after being kept 3

months in cans, had lost nearly all its sublimate. Lazarski, at his request, examined this question and found that sublimated gauze left exposed for 20 weeks lost ½ to ⅔ of its bichloride, and if boxed up for the same period ¼.

Finally, sublimate may be absorbed in fatally toxic amount. Its local caustic action—principally seen under sublimate dressings—has repeatedly caused eczema, erythema, urticaria, etc. Constitutional effects have been noted by several observers after such dressing, and after uterine injections. A few cases have ended fatally. M. refers to 6 at least, and gives one of his own, after extirpation of breast and axillary glands, where sublimate was only used in the dressing. It was the first case in which he used bichloride. The saw-dust dressing had been impregnated with 1 per cent. sublimate solution. Individual susceptibility varies.—*Northwestern Lancet.*

---

CROWN WORK.—*By M. W. Williams, Hopkinsville, Ky.*—Profitable and popular crown work must be done in short time, with ease, both to operator and patient, must be durable and artistic. This ideal has as yet not been attained by Howe, Bonwell, Richmond, Logan, or Sheffield, as many will attest. But that I have conquered part of the field, you are only to see to be convinced, by part of the field, I mean to say for incisors and canines can cry "*Eureka*"—I have complied with every condition mentioned above. I will give a short pen sketch, omitting details.

The crown is similar to old fashion wooden pivot crown, except the pivot-hole is lined with platina tube. A similar tube ¼ inch long, with bottom and depending shank of platina securely soldered to it designed to be secured in root canal. The lower portion of this tube is slightly larger than orifice. There is solid platina connecting post, in size easily to pass in tubes, in length so that mouths of tubes will meet when in position. The lower ¼ inch of post is slitted for reception of key wedge which is a small tapering steel wedge ¼ inch long and thickness of No. 1 file at its thickest end.

To adjust it, grind off root on line with gum. enlarge canal, so that tube and its depending shank will drop in loosely beyond level with end of root. Grind base of crown to approximate joint with end of root. Solder unslitted end of post in tube of crown, slip tube of root attachment over slitted end, mix agate cement thin, fill canal one-half full, force all in position, have the patient close the mouth, hold steady in articulating position until cement hardens; withdraw crown and post, leaving root attachment in exact position; trim down the cement, fill out with amalgam, allowing it to spread thinly, all over the end of root; insert small key wedge· place firm piece of wood on cutting edge of crown, with mallet gently drive it home; the end of the key striking the bottom of the tube will force itself in expanding sides of post to fill the dovetailed space. Thus you can readily see it is impossible for it ever to become loosened or detached. Advantages. Its simplicity, strength and durability; strongest because of the size and shortness of the post, and having more porcelain, strengthen by tub, in crown. The crown being out of the way. The ease of perfectly forcing amalgam round root attachment in canal, and efficiency in which the end of root can be protected from further decay: *It is self-articulating.* No exposure of cement, and but a line of amalgam at the gum margin. It is artistic in that it conceals art; can be permanently secured at one short sitting of thirty minutes without causing your patient any pain, and with the greatest comfort to yourself, knowing it will remain as long as the root stays in the mouth. One other advantage in case of accident to the porcelain, is the great ease with which another crown can be attached.—*Dental Register.*

---

DISCARD OXYPHOSPHATES.—*By C. A. Landrum, M. D.*—After about five years' close observation, I have arrived at the conclusion that the oxy-phosphates should be banished from operative dentistry. It will not do to cap exposed nerves, or to fill cavities in such close proximity to the pulp as to require a plastic material. I have carefully watched its action in the above character of cases, both in fillings of my own, and in those that have come to me from other dental practitioners. In the "setting" process, it would seem that sufficient phosphoric acid is set free to slowly but effectually devitalize the pulp, even though a considerable septum of dentine intervenes. This I suppose to be the action, although I have not as yet resorted to those chemical tests that would demonstrate it. This I do know: teeth exposed to its action, under the above conditions, invariably die after a few months; and they usually die painlessly. I have in many instances used it for this express purpose, where I did not wish to use arsenic. I notice several writers use it as a temporary stopping in sensitive cavities, for the cure of the sensitiveness, and I doubt not it is this very action which gives its application success. Arsenious acid will do the same, even more quickly, but thoughtful dentists would never use the latter agent for this purpose. Phosphoric acid is probably as severe a caustic as arsenic, and if used pure, as we do arsenic, would probably produce devitalization as surely and quickly. But the small amount that exudes from the inner surfaces of fillings is in such a state of dilution as to produce its results slowly. I know that many of the first men in our profession use it for capping nerves, for filling roots, for temporary fillings in sensitive cavities, for filling cavities with frail walls, and often as a lining under gold or amalgam. But I think if they will observe their own and others' work closely, they will come to this conclusion: That oxy-phosphate of zinc should only be used in dead teeth, or those we wish to destroy.—*Southern Dental Journal.*

---

NEW CAPSICUM PLASTER.—DR. FRANK B. DARBY, of Elmira, N. Y., has recently introduced a "Capsicum Plaster" to take the place of the "Capsicum bag"so successful used during the past year. Dr. Darby's improvement consists in placing the same ingredients as contained in the bag on a soft, flexible rubber-coated felt, of suitable size, forming a "plaster" which readily adapts itself and will stick to the gums and remain in position. Their use is indicated in all cases of pericemental inflammation, and pulp irritation, such as pain or tenderness about the roots of dead teeth, soreness caused by prolonged gold operations, wedging, or other causes. In fact, any tenderness or inflammation about the roots of teeth, is claimed to be relived by the prompt application of this plaster. Very favorable reports have been received from those who have tried both "bag"and plaster," and in all instances the plaster has been given the preference.

---

A NARROW ESCAPE.—"Dr. Theodore G. Lewis, the well-known dentist and editor of *The Dental Advertiser*, had a narrow escape in the burning building, and it is a noteworthy coincidence that he was one of the last to leave the *Commercial* building, when it was destroyed by fire in December, 1882. The Doctor thinks he has had his share of fire experiences in printing offices. Last evening he was in the job office of the *Express* on business. and was conversing with the foreman, when there was a sudden rush, and a cry of fire. The Doctor started down the stairway on the Exchange street side, but was driven back by the flames and smoke. He then recollected that a stairway leading to the roof was close at hand, and instead of going down he went up, reaching the roof in perfect safety, and remaining there for about three-quarters of an hour. Had he become confused, or been unfamiliar with the surroundings, he would doubtless have lost his life."—*Commercial Advertiser*.

